# ChIP-PaM: an algorithm to identify protein-DNA interaction using ChIP-Seq data

**DOI:** 10.1186/1742-4682-7-18

**Published:** 2010-06-03

**Authors:** Song Wu, Jianmin Wang, Wei Zhao, Stanley Pounds, Cheng Cheng

**Affiliations:** 1 Department of Biostatistics, St. Jude Children's Research Hospital, 262 Danny Thomas Place, Memphis, TN 38105, USA; 2 Bioinformatics Center, St. Jude Children's Research Hospital, 262 Danny Thomas Place, Memphis, TN 38105, USA

## Abstract

**Background:**

ChIP-Seq is a powerful tool for identifying the interaction between genomic regulators and their bound DNAs, especially for locating transcription factor binding sites. However, high cost and high rate of false discovery of transcription factor binding sites identified from ChIP-Seq data significantly limit its application.

**Results:**

Here we report a new algorithm, ChIP-PaM, for identifying transcription factor target regions in ChIP-Seq datasets. This algorithm makes full use of a protein-DNA binding pattern by capitalizing on three lines of evidence: 1) the tag count modelling at the peak position, 2) pattern matching of a specific tag count distribution, and 3) motif searching along the genome. A novel data-based two-step eFDR procedure is proposed to integrate the three lines of evidence to determine significantly enriched regions. Our algorithm requires no technical controls and efficiently discriminates falsely enriched regions from regions enriched by true transcription factor (TF) binding on the basis of ChIP-Seq data only. An analysis of real genomic data is presented to demonstrate our method.

**Conclusions:**

In a comparison with other existing methods, we found that our algorithm provides more accurate binding site discovery while maintaining comparable statistical power.

## Background

Understanding of transcriptional regulation mechanisms is of fundamental importance to the study of biological processes such as development, drug response and disease pathogenesis [1]. Through modulation of gene expression patterns, the differentiation and function of cells are tightly controlled. The on/off switch of specific gene expression is one of the main modulating mechanisms and is mainly through the association and disassociation of transcription factors (TFs) with their target gene promoters. Therefore, revealing the mechanism by which transcription factors regulate their target genes is essential to understanding many important biological processes. Several methods have been developed to identify the TF-target gene interactions and to investigate how and why cells respond to different signals. One such method, chromatin immunoprecipitation (ChIP) on a chip (ChIP-chip), is based on a tiling-array platform in which genomic DNA oligomers from gene promoters are pre-fixed. The DNA fragments immuno-precipitated from cell lysate by a TF antibody hybridize with the ChIP-chip array and TF-binding regions are identified by their high-intensity signals. Like all other array-based methods, however, this method can detect only targets included on the array.

More recently, with the advance of next-generation sequencing (NGS) technologies, ChIP-Seq has come into a wide use for transcription factor binding sites analysis. By directly sequencing the DNA fragments immunoprecipitated in a ChIP experiments, ChIP-Seq offers whole-genome coverage and greater sensitivity than the traditional ChIP-chip assay [2]. Several analytic algorithms have been proposed for ChIP-Seq data, including ERANGE [3], FindPeaks [4], MACS[5], SISSRs[6], CisGenome [7], QuEST [8], Useq [9], SPP [10], PeakSeq[11], BayesPeak [12], and GLITR [13]. Most of these algorithms aim to identify genomic regions enriched with ChIP-DNA fragments by using some negative control samples to remove/normalize some of the background noise from experimental procedures. For example, *Robertson et al *[2] identified the enriched regions by detecting peaks on a tag density map, generated by extending each mapped tag in the 3' direction to the average length of the DNA fragments in the sequenced DNA library. The signal map is the integer count of the number of overlapping DNA fragments at each nucleotide position. The control sample was generated by another ChIP-Seq experiment using the same antibodies against the un-stimulated cells, in which the transcription factor of interest is inactive and located in cytoplasm. *Rozowsky et al *[11] used the same idea of signal map, but used the raw input DNA as the control sample. *Chen et al *[14], studied a group of 13 transcription factors in E14 mouse ES cells by using a control sample obtained from another ChIP-Seq experiment with an irrelevant antibody, anti-GFP.

Although these negative controls are useful, they cannot account for an important source of noise signal - nonspecific DNA binding by TFs. This noise signal is difficult to control, as TFs must nonspecifically bind to DNA in order to efficiently access their unique binding sites among billions of nucleotides. Studies directly probing transcription factor dynamics at the single-molecule level in a living cell showed that TFs spend as much as 90% of their time non-specifically bound to and diffusing along DNAs [15]. Like the ability to bind to specific targets, non-specific binding to DNA is a *bona fide *TF ability and therefore, this type of noise signal cannot be eliminated by using a negative control. A few algorithms, such as SISSRs, MACS and FindPeaks, can identify transcription factor binding targets solely on the basis of ChIP-Seq data, without the use of control samples. However, with the exception of SISSRs, these algorithms identify binding sites merely by the number of tag counts within a genomic region, ignoring the forward- and reverse- strand information. SISSRs utilizes a logic rule in which the sequenced forward and reverse strands should lie separately on the two sides of the binding site; therefore, the difference between the forward and reverse tag counts would change sign on the binding site. The SISSRs algorithm usually generates significantly more binding sites than other algorithms [6], but these may include many false discoveries, as will be shown in later section.

Here we describe a new algorithm that incorporates the forward- and reverse-strand information but employs it differently. Our algorithm, ChIP-PaM, is based on peak counts modeling and pattern matching of a specific tag count distribution of forward and reverse strands generated by protein-DNA binding, followed by *de novo *motif finding and searching within the potential binding regions. We show that our algorithm can greatly reduce false positive findings while maintaining or improving accuracy and statistical power for binding site discovery.

## Results

### ChIP-PaM: Scoring the Enriched Genomic Regions in ChIP-Seq Data

**Figure 1:**
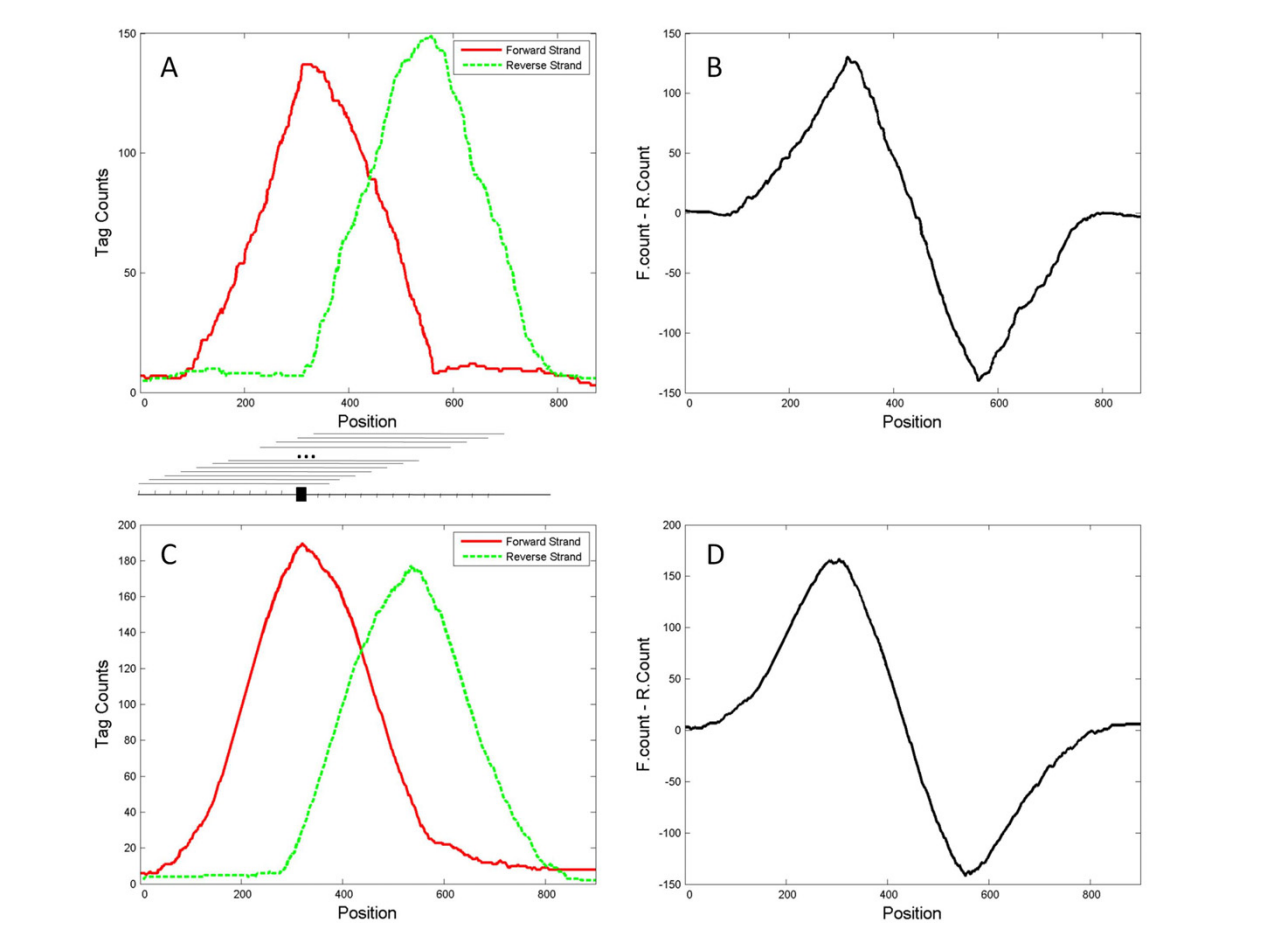
**Tag count distributions from the simulated and real genomic data**. **A**. Simulated forward- and reverse-strand count distribution for a region containing one TF binding site; **B**. Difference between forward- and reverse-strand tag counts shown in panel A. **C**. Forward- and reverse-strand tag count distribution in an example genomic region (from the real data application); **D**. Difference between forward- and reverse-strand tag counts shown in panel C.

TF binding sites (TFBSs) usually contains a short consensus binding site (CBS) sequence, ~ 10-20 base pairs, that provides target specificity. Suppose that there is one TFBS in a small genomic neighbourhood (*e.g.*, < 500 bp). In a ChIP-Seq experiment, ideally all forward-strand tags related to this TFBS should lie on the 5' side of the TFBS, and all reverse-strand tags should lie on the 3' side (Figure 1A), because only fragments containing the TFBS are pulled down for sequencing. Hence, given that the maximum fragment size selected for sequencing is *d*, a quantity known from ChIP-Seq experimental procedures, it is expected that the region beginning *d *bp upstream of the TFBS and ending at the TFBS will contain the greatest number of forward-strand tags, and the region beginning at the TFBS and ending *d *bp downstream of the TFBS will contain the greatest number of reverse-strand tags. If a potential TF binding region is scanned base pair by base pair with a sliding window of width *d *and the unique forward and reverse tags within the window are counted separately, the tag densities formed from forward and reverse strands will show a pattern of peak shift along the scanned genomic region, with the peak of one strand corresponding to the background signal of the other strand (Figure 1A, C). The tag counts at the peak position and the pattern of peak shift can be used as physiological evidence for a TF binding. Therefore, several sequences that show good evidence of containing the potential CBS can be aligned for *de novo *motif finding [16-19].

**Figure 2:**
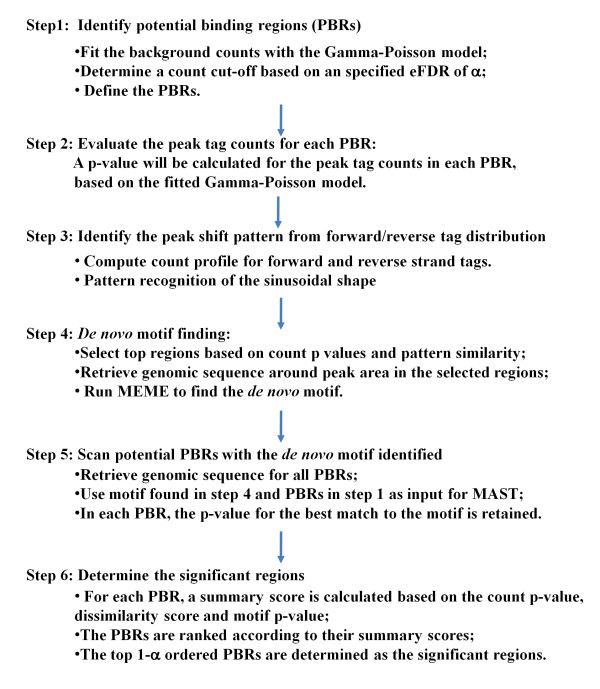
**Sequence the ChIP-PaM algorithm**.

Based on the above, we propose a new algorithm, ChIP-PaM, for ChIP-Seq data analysis. The method combines the tag counts at the peak position, the pattern recognition of the forward and reverse tag shift, and *de novo *CBS finding. It consists of six steps that are summarized in Figure 2 and described in detail below:

1. Identify potential binding regions (PBRs) by using a pre-specified empirical False Discovery Rate (eFDR): The whole genome is divided into non-overlapping regions *d *bp in size and the unique tags in each region are counted. The frequencies of the tag counts are then tabulated and fitted to a Gamma-Poisson (G-P) mixture model that can accommodate the over-dispersion of the data. The G-P model showed better fitting to the real data than the frequently used Poisson model and has been used in other software (*e.g.*, Cis-Genome [7]). The eFDRs for different tag count thresholds, defined as the ratio of the theoretical number of regions exceeding the thresholds by chance from the G-P model to the number of observed regions exceeding the same thresholds [6], can be calculated as the following

where k is a count threshold, Pr(count > k) is the probability of a tag count exceeding k in the G-P background model, N_total _is the total number of regions in the whole genome and N_observed_(count > k) is the number of observed regions with count exceeding k. A tag count cut-off corresponding to the pre-specified FDR rate (α, *e.g. *0.5) is then determined, and the genomic regions with tag counts greater than the cutoff are selected, and merged if adjacent, to form PBRs. The majority of the background tag signals are eliminated in this step, which can save immense amount of computing time in the following steps.

2. Evaluate the peak tag counts for each PBR: A sliding window of width *d *is used to scan the PBRs base pair by base pair and the forward and reverse tags within each window are counted to yield the forward and reverse tag distributions similar to Figure 1A and 1C. On the basis of the fitted G-P model, a p-value is calculated for the tag counts at the peak position within each PBR.

3. Identify the peak shift pattern from forward- to reverse- strand tag distributions: If a PBR contains a true TFBS, the difference between the forward- and reverse- strand tag counts will show a sinusoidal shape (Figure 1B, D). This shape is used for the pattern match and is identified by pattern recognition that employs a wavelet-based smoothing technique (see Methods for details). Dissimilarity scores comparing each PBR to a simulated reference pattern (S_R_) are computed and ranked.

4. *De novo *motif finding: PBRs with high peak counts and good sinusoidal pattern of forward to reverse tag count shift are considered as high-quality PBRs, *i.e.*, those most likely contain a TF binding site. The peak positions of the forward- and reverse-strand count distribution within the high-quality PBRs are obtained, and the genomic sequence a few bp (*e.g.*, 20 bp) upstream and downstream of the peak sites are retrieved to search for the *de novo *motif to which the TF might bind. Existing efficient algorithms such as MEME [18] are used for motif finding. A motif search algorithm typically generates several motifs, but only those contained by at least 25% of the input sequences are candidate motifs. Notice that each input sequence is only about 40 bp long; therefore it is reasonable to assume that each sequence contains either one motif or no motif.

5. Scan potential PBRs by using the *de novo *motif identified: A scoring matrix formed on the aligned consensus sequences identified in step4 is used to screen and score all PBRs identified in step 1. The smallest p-values corresponding to the best match to the scoring matrix in each PBR are retained as the PBR motif p-values.

6. Determine the significant regions: The PBR peak tag count p-values, pattern dissimilarity scores, and motif p values are integrated to re-rank the PBRs. Because the minimal p-value for the peak tag count may be as low as 10^-30^, whereas the minimal motif p values may be only 10^-5^, the scale difference for different score distributions is normalized. The p-values are log-transformed, rescaled to the same level and averaged to re-rank the PBRs. Finally, the top (1- α) re-ordered PBRs are selected as the significant TF target regions.

### Characteristics of the ChIP-Seq data

To illustrate our method, we used a public ChIP-seq dataset (GSE12782) deposited at the GEO by Rozowsky *et al *[11]. This dataset was originally analyzed by using PeakSeq. Several characteristics of the dataset are worth noting: 1) The raw input DNA is used as control; 2) The experiments were done in female Hela S3 cells; 3) The mitochondrial chromosomes (Chr M) were retained for sequencing; and 4) the transcription factor studied was STAT1, a TF with many well-known downstream target genes. These characteristics, while are not necessarily useful for the data analysis, provide a good opportunity to assess our proposed method, including an estimate of its false negative and false positive findings, as described below.

In the ChIP-Seq sample, ~26.7 million unique reads were mapped to the reference genome (hg18/NCBIv36, UCSC genome browser), and in the input sample ~23.4 million unique reads were mapped. The summary statistics are shown in Table 1. The genome-wide coverage for the ChIP sample is 0.015 read/nt. This low sequencing coverage is typical of ChIP-Seq data because the high-affinity of TF-DNA binding averts the need for deep sequencing on the whole genome. However, the sequenced fragments in ChIP sample and the input fragments in the control sample have very little overlap (0.75% of all sequenced tags, Table 1). This factor could be problematic if the input sample is used as local control, because the majority of fragments sequenced are present only in the ChIP sample or the input sample and therefore, the input fragments cannot serve as a representative control for ChIP data.

**Table 1:** Summary statistics of the ChIP-Seq dataset.

	unique reads			Copy percentage				
**Chr**	**ChIP only**	**Input only**	**in both**	**ChIP only**	**Input only**	**in both**	**Total length**	**ChIP Read/nt**

1-22, X	24.3 M	20.9 M	0.34 M	53.3%	45.9%	0.75%	3B	0.015

Y	11 K	11.6 K	1.7 K	45.5%	47.6%	6.9%	54.7 M	0.0004

M	33	3144	16.5 K	0.17%	15.9%	83.9%	16.5 K	1.191

Shown are selected basic characteristics used in the application, comparing chromosomes 1-22 plus X (combined because they are in the 2-copy state), Chr Y, and mitochondrial chromosomes (M). Chr Y is absent from Hela-S3 cells and indicates sequencing/mapping errors; mitochondrial chromosomes are located in cytoplasm and serve as an internal control for the nuclear chromosomes.

The mitochondrial chromosomes have been deeply sequenced due to their high copy numbers. Most cells contain many mitochondria, and each mitochondrion contains several copies of Chr M. Thus, Chr M copies are much more abundant than nuclear chromosomes [20]. This phenomenon is observed in the example dataset; the coverage on Chr M is ~3000 times more than that on the nuclear chromosomes from the input sample. Because mitochondrial DNA are physically separated from the nuclear STAT1 proteins, they can be used as a reference to estimate the background noise from the experimental procedure, such as the residual input DNA left in the ChIP sample. The expected number of noise reads is estimated to be 5 million for genomic DNAs (Table 2). However, the ChIP experiment generated about 24 million total noise reads, suggesting that most of the background fragments in the ChIP sample come from other sources, such as the nonspecific binding of a TF to the genome. As discussed in the Introduction, this type of noise signals cannot be adequately resolved by using controls. This is one challenge that promoted us to develop an algorithm independent of control samples.

**Table 2:** Comparison of nuclear and cytoplasmic chromosomes.

		Total Reads		
Chr		In ChIP	In input	ChIP/input ratio

	Specific Reads	2356286	441471	5.3373
	
1-22, X	Noise Reads	24285371	22585024	1.0753
	
	E(background	4959671		
	noise) in ChIP*	(0.2042)		

M		89835	409136	0.2196

*Assuming that reads mapped to mitochondrial chromosomes (M) are due to the experimental procedure and not to the non-specific binding of STAT1, the ChIP/input ratio for this background noise is 21.96%. Among chromosome 1-22 and X, the expected background noise in ChIP would be 22585024*0.2196 = 4959671, which accounts for 20.42% of total noise reads.

In contrast to Chr M, chromosome Y (Chr Y) is another extreme with very low coverage, because the male Y chromosome is absent in the female Hela S3 cells. Therefore, any enriched regions identified on the Chr Y should be considered as false positives. The fact that some reads were mapped to Chr Y in the dataset suggests there were mapping/sequencing errors. The reads mapped to Chr Y cannot be explained by sequence homology to chromosome X, because only unique reads were mapped to the reference genome. For these reasons, Chr Y serves as a perfect internal negative control. As shown in Table 1, 12.7 thousand of the 24.3 million ChIP reads were mapped to Chr Y. Given that the length of Chr Y is 54.7 M and the whole genome is about 3 billion bps, at least 0.9 M (3.64%) reads in the ChIP sample are predicted to be wrongly mapped or sequenced.

### STAT1 Targets Identified by ChIP-PaM in the ChIP-Seq Dataset

**Figure 3:**
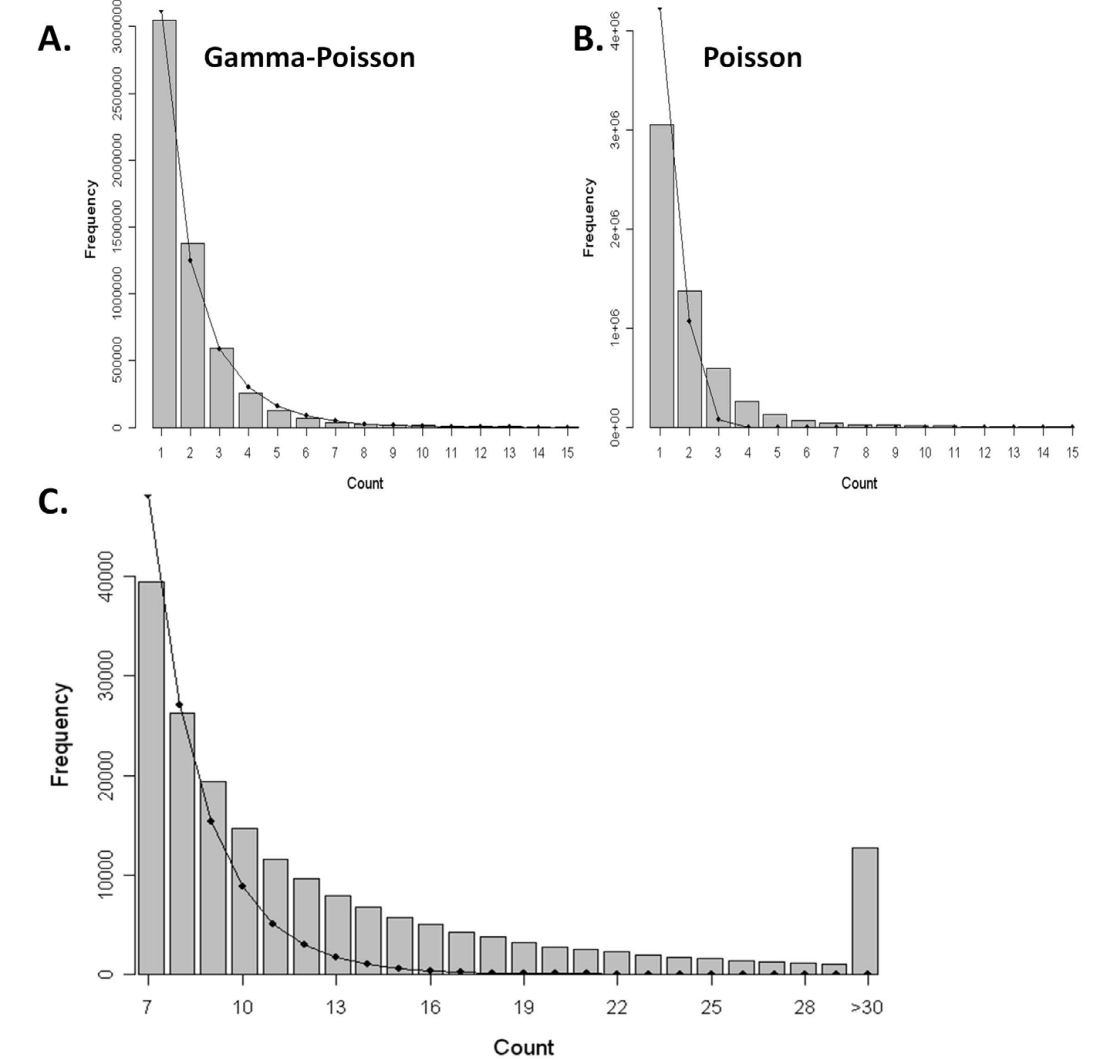
**Model fitting of the genome-wide tag count histogram**. **A**. Data fitted by the Gamma-Poisson model; **B**. Data fitted by the Poisson model. **C**. The detailed right-tail fitting by the Gamma-Poisson model.

We applied ChIP-PaM to the STAT1 ChIP-Seq dataset to examine the performance of our algorithm. From the experiment procedure, the maximum fragment size was known to be 250 bp. The whole genome was then divided into non-overlapping regions of 250 bp and the unique tags in each region were counted and tabulated into a tag count histogram. This histogram resembles an empirical distribution of the background noise count within a 250-bp region and was fitted by the G-P and Poison models (Figure 3A, B). The comparison between the two models showed that the G-P model fits the data much better than the Poisson model, suggesting significant dispersion of the data. Although almost 98% of the 250-bp windows contained six or fewer unique tag reads, a close look at the tail of the histogram and the fitted G-P density (Figure 3C) revealed significant tag enrichment in some regions; the eFDRs for different tag count cut-offs were calculated from these data.

With a pre-specified eFDR level of 0.5 for the count data, regions containing six or fewer unique tag reads were eliminated, leaving 69,809 PBRs. For each PBR, a p-value based on the count at the regional peak was calculated from the fitted G-P model, and a dissimilarity score based on shape pattern matching was computed. These two values were used to select 190 regions with low count p-values and the best-matched shapes, which showed strong evidence of TF bindings. Short genomic sequences around the peak sites (± 20 bp) in the 190 region were retrieved for *de novo *motif finding by using MEME. Because each input sequence was very short (40 bp), we specified that each sequence contained either one motif or none. A motif was identified in 112 out of 190 regions (58.9%), and all of the PBRs were then scanned for this motif by using the MAST program [18]. The *de novo *motif found strongly matched the STAT1 GAS motif previously identified and validated in biological experiments [2]. From the MAST scan, a p-value for the best motif match was obtained for each PBR. Therefore, three values were associated with every PBR: a p-value based on count distribution (p_c_), a dissimilarity score based on shape pattern matching (d_p_), and a p-value based on motif matching (p_m_). The three scores were log-transformed and the log-d_p _and log-p_m _were scaled to the level of log-p_c _by regression. The PBRs were then re-ranked by averaging the three scores. Because the original eFDR was specified to be 0.5, this suggests the true positive rate is also 0.5 in all PBRs. Therefore, the top 34905 regions are considered significant target regions.

The pre-specified eFDR level is somewhat arbitrary. A general rule in choosing the eFDR is that it should be large enough to incorporate sufficient number of PBRs for re-ranking, yet not too large to include too many noisy regions. When we used another α of 0.7 to analyze the data, 117,479 PBRs were identified and 35243 (117479*(1-0.7)) were selected as significant regions. The number of significant regions resulting from the two eFDRs is almost identical, as although a higher eFDR (α) yields a larger pool of PBRs, a smaller rate of true discovery rate (1-α) offsets the initial large number in the final result. We found that an eFDR of 0.5 is a good choice because it can generate sufficient PBRs for further improvement while being computationally more efficient than higher eFDRs.

### Comparison with Other Algorithms

We compared ChIP-PaM with SISSRs, PeakSeq and ChIP-PaM using tag counts information only. In the STAT1 ChIP-Seq dataset described above, PeakSeq identified 36,998 significant regions [11] and SISSRs identified 85,892 significant regions. To make the further comparison fair, we used the same number of top 36,998 regions for all algorithms.

**Figure 4:**
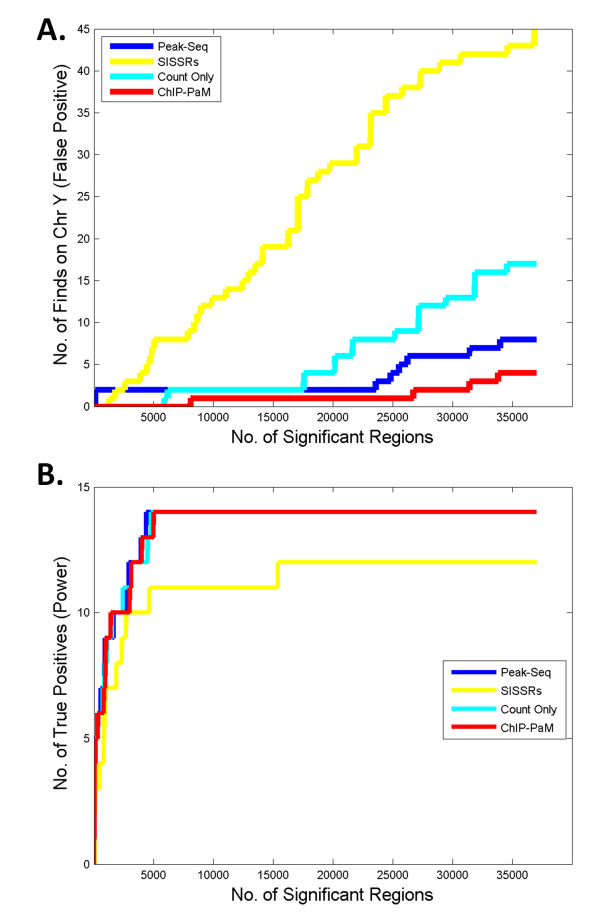
**Comparison of ChIP-PaM with SISSRs and PeakSeq and ChIP-PaM using count data only**. **A**. The number of findings on Chr Y is used to compare false positive findings. **B**. Fourteen known STAT1 GAS target genes are used to compare the true positive findings (*i.e.*, power).

The false discovery rate and power are the two most important criteria in assessing a model or algorithm. Although we do not know all of the true STAT1 binding sites in this dataset, we do have partial knowledge to make the assessment. As mentioned before, because Hela-S3 cells are female cells, any significant regions found on Chr Y should be spurious. Therefore, we used the number of findings on Chr Y as a surrogate for false discoveries. Figure 4A shows the "cumulative incidence" of findings on Chr Y as a function of the total number of significant regions. ChIP-PaM identified markedly fewer false positives than SISSRs and PeakSeq. Compared with ChIP-PaM using the counts data only, the incorporation of additional information about the tag distribution shape and motif score in ChIP-PaM significantly reduced the false-positive findings.

Twenty-two genomic promoters have been experimentally validated to be regulated and bound by STAT1 protein upon IFN-γ stimulation [2]. We used this information to compare the power of the algorithms. As shown in Figure 4B, PeakSeq, ChIP-PaM and ChIP-PaM using count only have almost identical "cumulative power"; they all detected a maximum of 14 of 24 positive promoters. However, the SISSRs had the least power to detect the known sites. In the rest 8 targets that were not identified by either method, a detailed look at their genomic regions found that essentially no reads were mapped in this ChIP-Seq sample, and therefore no algorithm can detect them. This suggests that ChIP-PaM is efficient in identifying the true STAT1 targets.

**Figure 5:**
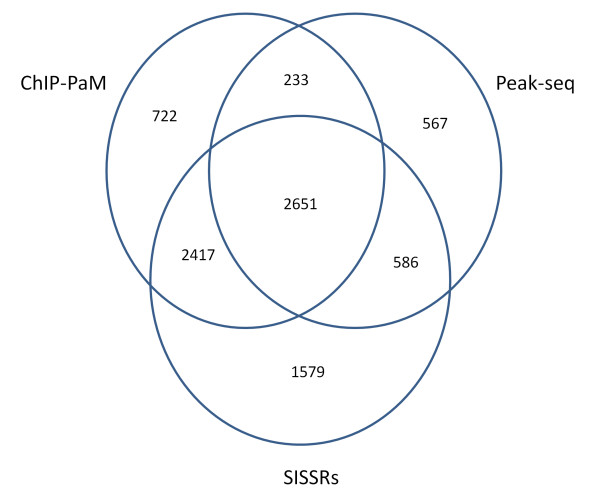
**Venn Diagraph of genes found by the three algorithms**.

**Figure 6:**
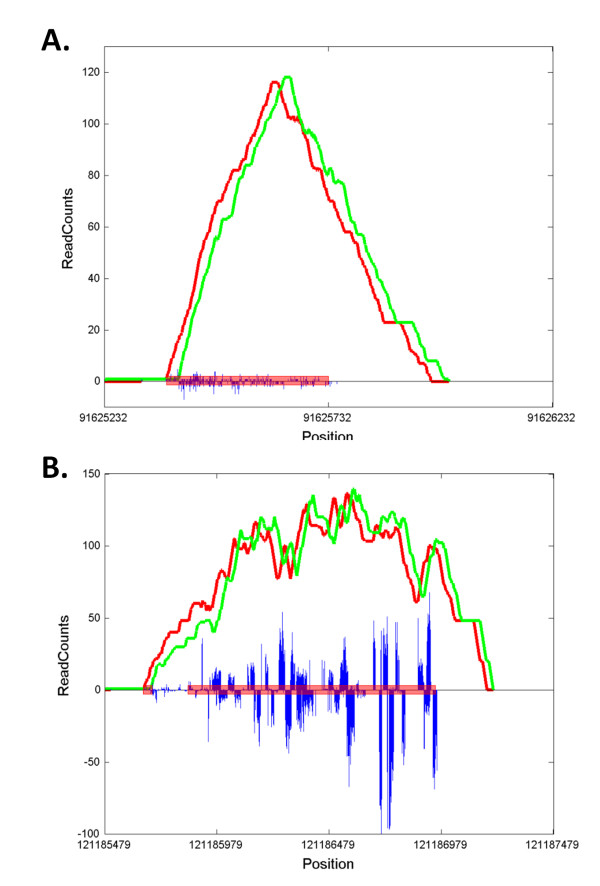
**Examples of two real genomic regions that are identified as significant by SISSRs but not by ChIP-PaM and PeakSeq**. **A**. Chr1: 91625233- 91625750; **B**. Chr1: 121185480- 121186959. The red line represents forward-strand counts and the green line represents reverse-strand counts. The positive blue bars represent forward tag reads and the negative blue bars represent reverse tag reads. The broad pink line delineates regions identified as significant by SISSRs.

We used the RefSeq to annotate the significant regions found by the three algorithms. If an identified STAT1 binding region is located within -250 bp to 5 kp from a gene's transcription initiation site, the gene is considered a STAT1 target. The target genes found by the three algorithms share close similarity (Figure 5), and 2,651 of them were identified by all three methods (Additional file 1). For the 2,651 common genes, the rank correlation was 0.71 between ChIP-PaM and PeakSeq, 0.55 between ChIP-PaM and SISSRs, and 0.52 between PeakSeq and SISSRs. These data suggest that ranking by ChIP-PaM is more similar to ranking by SISSRs, since both used a pattern of forward and reverse tags. However, the pattern utilized by SISSRs is somewhat too local as it captures, within a small region, the sign change of the difference between the forward and reverse tag counts. This fact may explain why SISSRs yields a much higher false positive rate. Two genomic examples on chromosome 1, chr1: 91,625,233 - 91,625,750 (Figure 6A) and chr1: 121,185,480 - 121,186,959 (Figure 6B), are shown to illustrate the point. These two regions were identified as significant by SISISRs, but not by either ChIP-PaM or PeakSeq. The overall regional pattern clearly indicates that the tag enrichments in these two regions are not caused by TF binding; however, the rapid local sign change of the difference between the forward and reverse tag counts causes SISSRs to consider them as significant.

## Discussion

With the advance of the next-generation techniques, ChIP-Seq experiments are expected to be in great demand for the important biological studies of transcription regulatory network. Therefore, more efficient models and algorithms to analyze such data are urgently needed. Here we have proposed a new method of analysis of ChIP-Seq data that is based on ChIP-Seq sample only and that retains and even improves the accuracy and statistical power of binding site discovery.

There are four potential major mechanisms by which a region with enriched tags might be observed in ChIP-Seq data: (1) "true positive" TF target regions; (2) focal amplification of certain genomic regions; (3) nonspecific binding of TFs to the genome; and (4) random noise from experimental procedures. The difficulty arises in effectively separating tag enrichment resulting from (1) from those resulting from the others. Our algorithm is designed to improve the accuracy of finding in each instance. The Gamma-Poisson modeling takes account of the non-uniform noise background across the genome and helps to model both nonspecific TF binding and random noises. The use of pattern recognition to match a TF binding pattern will improve the detection of the true enrichment pattern. For example, enrichment induced by focal amplification shows a shape pattern (similar to Figure 6A) very different from that of enrichment by TF binding, and the pattern matching step of our algorithm can efficiently remove it from the final results. Furthermore, the *de novo *motif finding and searching step will help eliminate non-specific binding regions that do not contain the conserved motif sequences.

Because our proposed method incorporates three lines of evidence to determine the significant TF target regions, it is more robust than other current methods and detects fewer false positive bindings. However, it is often difficult to determine the statistical accuracy of the findings when multiple lines of evidence are integrated. In ChIP-PaM, we propose a novel data-based two-step FDR procedure to solve this problem. In this procedure, an eFDR (α) derived from the genome-wide tag count distribution is pre-specified to select the potential TF binding regions, and then the shape and motif information is incorporated to re-rank the selected PBRs. As the α level controls the overall FDR and re-ranking of the PBRs will not change it, the top (1- α) re-ordered candidate regions are considered to be significant. This data incorporation procedure can be potentially applied to other integrated analyses as well. Another advantage of our algorithm is that unlike other methods, in which the average length of the ChIP fragments must be estimated, ChIP-PaM makes use of the maximum length of the fragments, which is known from the experimental procedures. This should reduce the variation of the findings.

The entire analysis of the STAT1 example dataset took about 1.5 hours on a regular desktop computer. Therefore, our algorithm is computationally efficient. The majority of time is spent on the shape matching and motif identification steps. Only 5 minutes is needed to scan the PBRs and compute preliminary result from them. If the inference is to be made on the basis of the count data and only the genome scanning part is required, our algorithm would be extremely fast and might be algorithmically attractive for other applications, such as epigenetic analysis of modified histone proteins [21]. Further investigation is needed in this direction.

Finally, one point worth noting is that although our algorithm requires no control sample, control samples may have an important role, depending on the scientific questions asked. For example, if a study were to compare the binding site difference of a TF in its "active" *v.s. *"inactive" form, a ChIP-Seq sample for the "inactive" TF would be a perfect control. In this case, two ChIP-PaM analyses would be performed independently on the two ChIP-Seq samples.

## Conclusions

We propose a new algorithm, ChIP-PaM, for genome-wide identification of the transcription factor target genes by using the ChIP-Seq data. Unlike other methods of analyzing ChIP-Seq data, ChIP-PaM incorporates three lines of evidences, including tag count modeling at the peak position, pattern matching of a specific tag count distribution, and *de novo *motif finding and searching along the genome. A comparison with existing methods showed that our method can greatly improve the accuracy of binding site discovery while maintaining comparable statistical power.

## Methods

### Sequencing Procedures

Because information from sequencing procedures is used as parameter inputs of our algorithm, we will briefly describe the sequencing process. In ChIP-Seq sample preparation, genomic DNAs are either sonicated or digested into random fragments and size-selected (100-800 bp) for better sequencing accuracy. The ChIP-DNA fragments submitted for sequencing are therefore within a certain range of length, e.g. 150-250 bp. Owing to cost and technical reasons, typical tag reads acquired from sequencing apparatus for ChIP-Seq experiments are small (~30-60 bp), and consequently reflect only the two ends of the fragments, not the whole fragments. The end positions of fragments can be revealed by mapping the short-read tags back to a reference genome. For single-end sequencing, because the sequencing adaptors are ligated onto two ends of a fragment randomly, approximately equal numbers of tags are expected to be obtained in forward and reverse directions within a region.

### Gamma-Poisson Model

If the tag reads from ChIP-Seq experiments are uniformly distributed on the genome that is divided into equal windows of *d *bp, basic probabilistic considerations imply that the distribution of unique tag counts in a certain window should obey the Poisson distribution. However, since the binding affinity of a TF to the whole genome is not uniform (*i.e.*, specific and non-specific bindings), the ideal Poisson model will not be followed. It is expected that the whole count data will be a mixture of Poisson distributions. Assuming that the majority of DNA fragments are background signals, and that the significantly enriched regions reside largely in the right tail of the distribution, a zero-truncated Gamma-Poisson density can be employed as the background model for the count data. The Gamma-Poisson model is shown as below:

Poisson probability mass:

Gamma density function:

Gamma-Poisson (G-P) mixture density function:

Zeros are not included in the model, as zero counts can occur simply because of non-mappability of certain regions and therefore does not truly reflect the zero counts in the model. The G-P model is employed to infer whether a specific region has a significantly high count. The mean count r(1-p)/p is positively correlated with the size of the window, *i.e.*, the maximum fragment size *d*, and the sequencing depth of total number of reads obtained, and r(1-p)/p^2 ^measures the dispersion of the data. There are several ways to estimate the model parameters [22]. Here we employ the maximum likelihood method. The maximum-likelihood estimate of the parameters (r, p) was done by using the nonlinear optimization algorithms implemented in R [23].

### Pattern Matching with Wavelet Smoothing

The pattern of peak shift in Figure 1A represents the difference in the tag count distributions between the forward and reverse strands. Theoretically, this difference has a sinusoidal shape, as illustrated in Figures 1B. The observation patterns S_PBR _are calculated for all the PBRs that pass the initial genome scan. A reference pattern (S_R_) can also be generated from the simulation of a ChIP-Seq experiment. The S_PBR _and S_R _can then be compared to calculate the dissimilarity score. To remove noise signal from the data, we perform wavelet smoothing on S_R _and S_PBR _by using maximal overlap discrete wavelet transform with la8 wavelet filter and hard thresholding [24]. The locations of the peaks and troughs of S_R _and S_PBR _are then matched by translation and scaling. The maximum amplitudes of both S_PBR _and S_R _are scaled to 1. Assuming the total length of the reference strand is n, the dissimilarity score is defined as the sum of the absolute differences between S_PBR _and S_R_.

### Simulation of a Reference Pattern

A TF ChIP-Seq experiment is simulated to generate the reference pattern used for the pattern matching. Suppose there are 3 × 10^6 ^DNA fragments with random length between 150 and 250 bp that are uniformly distributed on a 1 kb genomic region. Assume there is one TFBS located in the center of the region. Assume that the probability of being pulled down for sequencing is 0.1% for DNA fragments containing the TFBS, and 0.005% for fragments not containing the TFBS and that the two ends of one fragment have an equal probability of being sequenced. The tag count distributions for the forward and reverse strands along the genomic region are generated and used to build the reference pattern.

### Motif Finding and Searching

MEME is one of the most widely used tools for *de novo *consensus motif finding [18]http://meme.sdsc.edu/meme4_1/. Because MEME searches for motifs by performing Expectation-Maximization (EM) on a motif model, it is very time-consuming for large input datasets. We chose an input size of fewer than 500 sequences for motif finding, to minimize the drawbacks of too few sequences (not representative) or of a number too large to be computationally feasible. Only motifs contained in at least 25% of sequences submitted were considered to be canonical motifs and were used for subsequent motif searching. Also, as each submitted sequence for motif finding is approximately 40 bps, we expect that such short sequences will not contain more than one motif. Therefore, the maximum number of motifs in each sequence was set at 1 for MEME. Position-specific scoring matrices (PSSMs) generated by MEME were used as input for MAST, a sister program of MEME developed for motif alignment and searching. MAST compares the sequence from PESRs with the motifs identified from MEME and calculates match scores for each sequence. A p-value is generated to score the probability of each sequence that may contain the motif found.

## Competing interests

The authors declare that they have no competing interests.

## Authors' contributions

SW conceived the idea, wrote the R code, analyzed the real genomic data, and wrote the manuscript. JW wrote the Perl code to integrate the software. WZ conceived the idea of pattern matching with wavelet smoothing, wrote the code, and helped to write manuscript. SP and CC was involved in data analysis and helped to write manuscript. All authors have read and approved the final manuscript.

## Supplementary Material

Additional file 1 **The common genes identified by three algorithms**. The file contains the 2,651 common genes that are identified by all three algorithms: ChIP-PaM, PeakSeq and SISSRs, and also their relative rankings by each method. Click here for file
